# A study on the effects of interfering with the conventional sequential protocol for chemical isolation and characterization of chitosan from biowaste of giant freshwater prawn *Macrobrachium rosenbergii*

**DOI:** 10.1016/j.heliyon.2023.e13970

**Published:** 2023-02-22

**Authors:** Benedict Terkula Iber, Donald Torsabo, Che Chik, Fachrul Wahab, Siti Abdullah, Hassimi Abu Hassan, Nor Kasan

**Affiliations:** a Higher Institution Centre of Excellence (HICoE), Institute of Tropical Aquaculture and Fisheries (AKUATROP), Universiti Malaysia Terengganu, 21030, Kuala Nerus, Terengganu, Malaysia; b Department of Fisheries and Aquaculture, Joseph Sarwuan Tarka University, (Formally Federal University of Agriculture, Makurdi), P.M.B.2373, Makurdi, Benue State, Nigeria; c Department of Chemical and Process Engineering, Universiti Kebangsaan Malaysia, 43600, UKM Bangi, Selangor Darul Ehsan, Malaysia

**Keywords:** Chitin, Chitosan, Biowaste, Demineralization, Deproteinization, Deacetylation, *M. rosenbergii*

## Abstract

Unless better measures are put in place to address the environmental and social impacts emanating from the huge waste generated from sea food processing industries; ‘tragedy of the commons’ is inevitable. Needless to re-emphasise the enormous contributions of aquaculture as the perfect substitute to capture fisheries which has been proven unsustainable. Be that as it may, the huge amount of bio-waste produced could be transformed into useful products such as chitin and chitosan with far reaching applications. Chitin and chitosan have been consistently processed from many sources following the traditional chemical sequence of Demineralization (DM), Deproteinization (DP), Decolouration (DC) and Deacetylation (DA). In this study, this method was re-ordered, resulting to 4 sequences of chemical processes. HCl, NaOH, ethanol (97%) and NaOH (50%) were used for DM, DP, DC and DA respectively. The results of this study showed that better chitin (23.99 ± 0.61%) and chitosan (15.17 ± 1.69%) yields were obtained from sequence four (SQ4) following the order of DC-DM-DP-DA. In addition, physicochemical properties such as DDA (80.67 ± 2.52%) and solubility (66.43 ± 2.61%) were significantly higher (p ≤ 0.05) in SQ4 thereby making the obtained product suitable for use as coagulant and flocculant in wastewater treatment. Results of FTIR, XRD and SEM of the study proved that the resultant product exhibited the characteristic nature of chitosan with porous and fibril nature. In the analysis of the physical properties of chitosan obtained from bio-waste of *Macrobrachiumrosenbergii*, the high Carr's index (CI) and low bulk as well as tapped densities were an indication that the chitosan produced in this study had poor flowability and compressibility, thereby making it unfit for application in pharmaceutical industries.

## Graphical abstract

**Figure:**
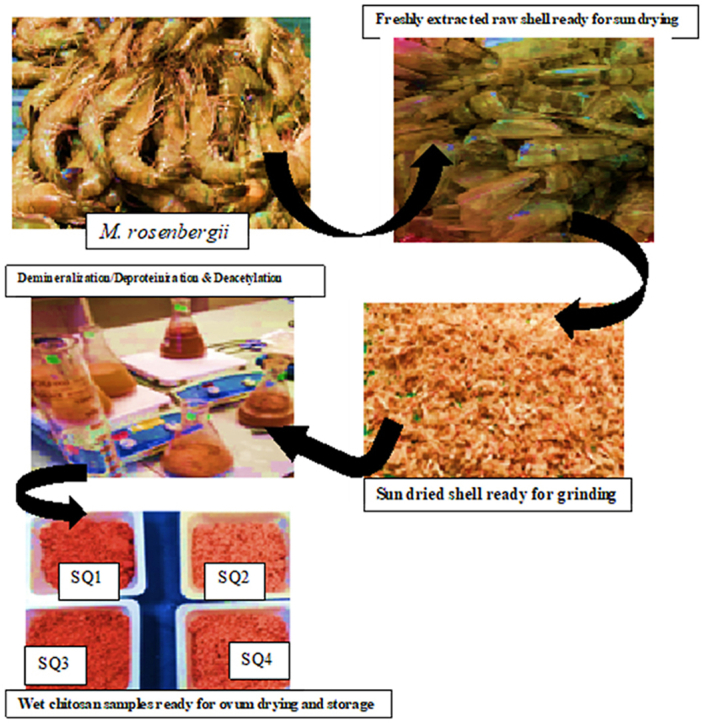

## 1 Introduction

The enormous impacts of wastes emanating from industries processing marine organisms have become a source of worry in recent years. These marine products are turned out in huge quantities without a corresponding recovery plan, thereby leading to environmental degradation and other social impacts [1]. Studies have shown that millions of tons of fisheries by-products find their way into the environment every year. These hazardous wastes are reportedly high in terms of biological oxygen demand (BOD), chemical oxygen demand (COD) and active in pathogen proliferation. It is worthy of note that these wastes, which are basically made up of proteins, minerals, pigments and chitin could be transformed into very useful product such as chitosan, with versatile applications in medicine, agriculture, food processing as preservative, tissue engineering, wastewater treatment [[2], [3], [4]].

Chitosan is the most important derivative of chitin. It is obtained through a deacetylation process from various sources including crustaceans, mollusc, fish scales, fungi and some algae [[5], [6], [7]]. Ref. [8] reported that crab and shrimp are so far the most commercially exploited species for chitosan production. The industrial extraction of chitosan from the reported sources begins with treatment of raw powder shell with acid to remove inorganic substances in a process called demineralization. This is shortly followed by deproteinization–a process where protein substances are removed by treatment with alkali. In most cases, pigments are removed by soaking in alcohol while deacetylation at high alkali concentration and temperature produces chitosan [9]. Due to its numerous and evolving properties such as non-toxicity, biocompatibility, eco-friendliness, chitosan has become a major sort after material in recent years [5]. Furthermore, the diversity in the natural origin as well as the high-level chemical and physical variation of chitosan have been reported to impact significantly on its utilization [5]. In addition, this variability in the physicochemical properties of chitosan has also been known to be affected by the method of chitosan preparation [10].

Over the years, the chemical method of chitosan extraction has been favoured against the biological method. This is largely because of the less complex nature and the potential for large scale production associated with the chemical method [11]. The most commercially exploited sources like crabs and shrimp as well as others such as cell wall of fungi, diatoms, exoskeleton of corals and sponges have successfully been utilized to produce chitosan through the traditional chemical method [[12], [13], [14], [15]]. The traditional chemical method of chitin and chitosan production which proceeds in the order of demineralization, deproteinization, decolouration and deacetylation has been utilized to obtain 17–32.2% chitin yield from the shell wastes of snow crab and the northern prawn; 14% chitin yield from blue crab; 17.8% from grey shrimp and 4.5–7% from speckled shrimp [9]. In another development, 82 ± 1.90%, 80 ± 1.45%, 78 ± 1.60% chitosan yield were obtained from the shell waste of prawn at 1.5, 3 and 6 h respectively using the traditional method. Several other authors have utilized this same method to isolate chitosan from diverse sources [3,[16], [17], [18]].

From the studies reviewed so far, there is no comprehensive study on isolation and characterization of chitosan from dry shell wastes of *M. rosenbergii*; especially on how the sequential steps affect the final product. Therefore, this study painstakingly assessed the extent of influence in re-ordering the traditional arrangement of the chemical isolation steps of chitosan. In addition, careful observations are also made of the effects on the physicochemical properties of the resultant chitosan from the various sequential arrangements, with an intent of making informed recommendations on the best sequential protocol.

## 2 Materials and methods

### 2.1 Collection and preparation of raw material

Fresh whole samples of *M. rosenbergii* were obtained from the fish wet market located in Terengganu, Malaysia. Fresh shells were thoroughly washed to eliminate dirt and oven dried at 70° to total moisture removal. Samples were later blended to powder using electric blender (Philips HR2118/01, 600 W, China).

### 2.2 Chemical preparation of chitin and chitosan

**Fig. 1:**
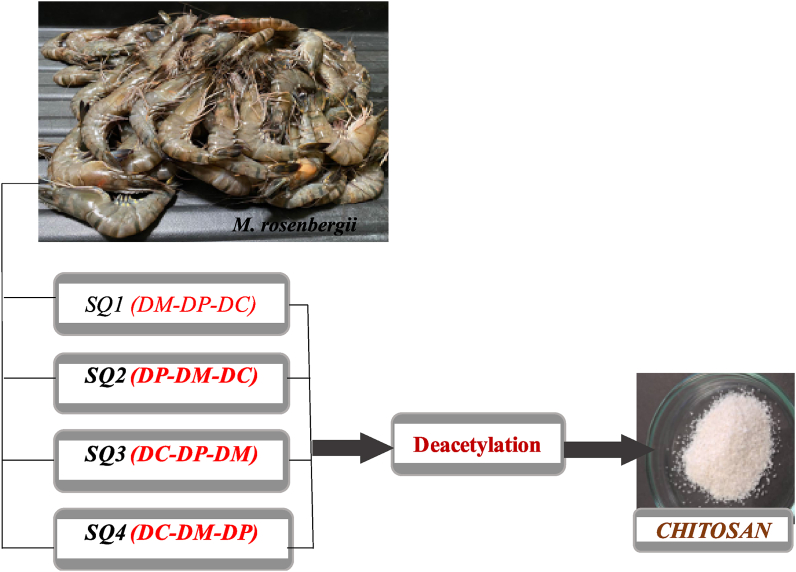
Schematic diagram showing the various sequences adopted for chitosan production.

Four samples of *M. rosenbergii* chitosan were produced and tagged as SQ1, SQ2, SQ3 and SQ4 representing sequence 1, 2, 3, and four respectively. The sequences were designed by re-ordering the traditional sequence of demineralization (DM), deproteinization (DP), decolouration (DC) and deacetylation (DA). In this arrangement, SQ1 which was considered as the control followed the traditional sequence of DM-DP-DC-DA [19]. Furthermore, SQ2, SQ3 and SQ4 followed DP-DM-DC-DA, DC-DP-DM-DA and DC-DM-DP-DA respectively as shown in Fig. 1. Determination of physicochemical properties [20] of isolated chitosan was then carried out and further comparisons among the sequences were carried out to identify which of them produced chitosan of better quality [20].

In the present study, 50 g of dry powder *M. rosenbergii* was used for the chitosan extraction. Determination of inorganic content (Demineralization) was achieved using 1 M of HCl which was added in the ratio of 1:15 (g/mL) at 50 °C and kept for 3 h s under 250 rpm agitation. Furthermore, the protein in the raw shell was removed (Deproteinization) by heating demineralized shell with 1 M NaOH, mixed at solid/liquid ration of 1:15 (g/mL) and kept at 90 °C under same level of agitation and time as in demineralization. At this stage, the neutral chitin was oven dried at 70° for 12 h and further decolorized using 95% ethanol, mixed at a mass/volume ration of 1:5 and kept at ambient temperature for 30 min. Finally, upon successful washing of decolorised chitin to neutral pH, the oven dried chitin was mixed with 50% NaOH at 1:15 (g/mL) and heated at 100 °C under 250 rpm agitation for 3 h s. Produced chitosan was washed to neutrality and oven dried at 70 °C for 12 hr.

### 2.3 Characterization of chitosan

#### 2.3.1 Chitin/chitosan yield, percentage moisture, ash content

The level of chitin in the shell of *M. rosenbergii* was estimated considering the difference in mass of powder shell after demineralization, expressed in percentage (Equation (1)). On the other hand, percentage chitosan yield was determined as the dry weight of residual mass of shell after deacetylation (Equation (2)) [21].

(1) $$Yield\;of\;chitin\;\left(\%\right)=\frac{Extracted\;chitin\;\left(g\right)}{Ground\;shell\;of\;M.\;rosenbergii\;\left(g\right)}\times 100$$

(2) $$Yield\;of\;chitosan\;\left(\%\right)=\frac{Extracted\;chitosan\;\left(g\right)}{Extracted\;chitin\;\mathrm{from}\;M.\;rosenbergii\;\mathrm{shell}\;\left(g\right)}\times 100$$

Determination of moisture level in the isolated chitosan was achieved through the gravimetric method by Official methods of analysis (AOAC) (2000) and [22]. In this process, 1 g of chitosan sample was oven dried at 70 °C to constant weight and moisture level was estimated as the weight difference, expressed in percentage as shown in Equation (3).

(3) $$Moisture\;\left(\%\right)=\frac{Wet\;weight\;of\;the\;sample\;\left(g\right)-Dry\;weight\;of\;the\;sample\;\left(g\right)}{Wet\;weight\;of\;the\;sample\;\left(g\right)}\times 100$$

Also in this study, a silica crucible containing 1 g of chitosan sample ignited in a muffle furnace set at 600 °C for a period of 5 h s and later placed in a desiccator after cooling. The estimation of percentage ash was carried out using the weight of crucible and remaining ash as shown in Equation (4).

(4) $$Ash\;content\;\left(\%\right)=\frac{Weight\;of\;the\;ash\;residue\;\left(g\right)}{Sample\;weight\;\left(g\right)}\times 100$$

#### 2.3.2 Determination of water binding capacity (WBC) and fat binding capacity (FBC)

This was determined using the approach described by Ref. [23]. 10 mL of distilled water was added to a centrifuge tube after 0.5 g of chitosan sample was tested for WBC. To dissolve the chitosan, the liquid was then vortexed for 1 min and allowed at room temperature for 30 min. The tube was then centrifuged for 25 min at 3200 rpm after shaking for 5 s every 10 min. The tube was again weighed to obtain the water bound after the supernatant was decanted. The following formula was used to determine the WBC: (Equation (5)) [23].

(5) $$Water\;binding\;capacity\;\left(WBC\right)\left(\%\right)=\frac{water\;bound\;\left(g\right)}{initial\;sample\;weight\;\left(g\right)}\times 100$$

Chitosan's FBC was calculated using a modified method by Refs. [21,23]. In order to measure FBC, a centrifuge tube containing 0.5 g of chitosan sample, 10 ml of oil (soybean oil), and 1 min of vortex mixing to disperse the samples was gauged. The mixture was centrifuged at 3000 rpm for 25 min after being left at room temperature for 30 min while being shaken for 5 s every 10 min. The supernatant was then tapped, and the cylinder was reweighed after that. The FBC was calculated using Equation (6) [21,23].

(6) $$Fat\;binding\;capacity\;\left(FBC\right)\left(\%\right)=\frac{Fat\;bound\left(g\right)}{Initial\;sample\;weight\;\left(g\right)}\times 100$$

#### 2.3.3 Solubility and DDA of sample

Chitosan's solubility in mild acidic solution was evaluated using a modified version of the [21,24] methods. To create 1% chitosan solution, 1 g of chitosan was dissolved in 1% acetic acid solution. This mixture was swirled with a magnetic stirrer at ambient temperature for 2 h. The mixture was then centrifuged at 600 rmp for 5 min, and then filtered through Whatman No. 1 filter paper that had been pre-weighed (Wi). The filter paper was reweighed after being further dried at room temperature (Wf). The formula below was used to compute the solubility percentage (Equation (7)) [21,24].

(7) $$Solubility\;\left(\%\right)=100-\left[\frac{wf-wi}{Ws}\times 100\right]$$

where;

Wi and Wf refer to initial and final weight of filter paper, while Ws is the weight of substance (chitosan).

For FTIR spectra analysis utilising an I.R instrument, c hitosan samples were produced in KBr discs and film (MB- 100, Bomem Hartmann & Braun, Quebec, Canada). After frequency was set to 4000-400 cm^−1^, DDA was calculated using the technique suggested by Ref. [25] and stated in Equation (8) [25].

(8) $$100-\left[\left(\frac{A1655}{3450}\right)\times \frac{100}{1.33}\right]$$

#### 2.3.4 Bulk density (BD), tapped density, compressibility, Hausner ratio (HR) and Carr's index (CI)

According to a method by Ref. [26], the bulk density (BD) of the chitosan sample was computed. Chitosan sample weighing 5 g was put into graduated centrifuge tube, and volume was recorded without shaking. To determine an average volume, this process was performed five times. Equation (9)'s formula was used to compute bulk density [26].

(9) $$Bulk\;density\;\left(g/mL\right)=\frac{mass\;of\;the\;sample}{V}$$

where V is the untapped volume of sample in the centrifuge tube.

Chitosan dry sample weighing 10 g was vortexed until a constant volume was produced in a calibrated centrifuge tube in order to determine the tapped density of the material. Across all samples, the experiment was conducted twice. In order to calculate tap density, Equation (10) was used.

(10) $$Tap\;density\;\left(g/mL\right)=\frac{mass\;of\;the\;sample}{Vtap}$$

where Vtap is the volume of the substance in the centrifuge tube after tapping or shaking.

In this investigation, the proportional variation in the volume of the substance in response to pressure changes or a change in mean stress was used to estimate the compressibility of dry powder chitosan. Equation (11)'s expression for the determination of compressibility was reached.

(11) $$Compressibility=\frac{100\left(Vo-Vf\right)}{Vo}$$

where; V_o_ is the unsettled apparent volume while V_f_ is the final volume.

The frictional forces between the particles of chitosan are shown by the HR of samples. This was made following the formula provided (Equation (12)).

(12) $$Hausner\;ratio\;\left(HR\right)=\frac{Dtap}{Dbulk}$$

where Dtap and Dbulk are the tap and bulk densities of the substance respectively.

**CI** refers to the cohesiveness of the chitosan particles and expresses the ability of the particles to aggregate. CI was obtained using Equation (13).

(13) $${Carr}^{\prime }s\;\left(CI\right)=\frac{Dtap-Dbulk}{Dtap}\times 100$$

where Dtap and Dbulk are the tap and bulk densities of the substance respectively.

#### 2.3.5 Percentage inorganic content

The level of inorganic content in the raw shell was determined using Equation (14).

(14) $$Percentage\;inorganic\;removal\left(\%\right)=\frac{mass\;before\;demineralization-mass\;after\;demineralization}{mass\;before\;demineralization}\times 100$$

#### 2.3.6 Percentage protein content

Percentage protein content in the *M. rosenbergii* shell was expressed as in Equation (15).

(15) $$Percentage\;protein\;removal\;\left(\%\right)=\frac{mass\;before\;deproteinization-mass\;after\;deproteinization}{mass\;after\;deproteinization}\times 100$$

#### 2.3.7 Percentage pigment

The percentage level of pigmentation in the shell was determined using Equation (16).

(16) $$Percentage\;pigment\;removal\;\left(\%\right)=\frac{mass\;before\;decoloration-mass\;after\;decoloration}{mass\;before\;decoloration}\times 100$$

#### 2.3.8 X-ray diffraction

To determine the crystallinity of the chitosan, wide-angle X-ray diffraction investigations were performed using a diffractometer XRD (Bruker model D8 ADVANCE), operating at a voltage of 40 V and a current of 30 mA with Cu K radiation (= 1.54060). The XRD pattern was captured in a fixed time mode at room temperature in the 2θ range of 9–80°.

#### 2.3.9 Scanning electron microscope (SEM) and morphological analysis

Using a scanning electron microscope, morphological study of the pulverized chitosan surface was done at a 1000× resolution (JEOL, JSM-7600 F, Japan). Samples of chitosan were examined for thickness, shape, and form [11].

#### 2.3.10 Fourier transformed infrared spectroscopy (FTIR)

After samples were made in KBr discs and film, infrared spectra of the chitosan samples were acquired using an I.R equipment (MB-100, Bomem Hartmann & Braun, Quebec, Canada). The range for frequency was 4000–400 cm^−1^ [27].

### 2.4 Statistical analysis

The SPSS statistical package programme (SPSS 22.0. for windows, SPSS Inc., Chicago, IL USA) was used to analyse the data from this investigation. The arithmetic mean standard deviation was used to express the findings of batch tests that were carried out in triplicate. One-way ANOVA and Tukey HNK were used to examine the statistical significance of the mean differences at the significant threshold of p ≤ 0.05.

## 3 Results and discussion

**Fig. 2:**
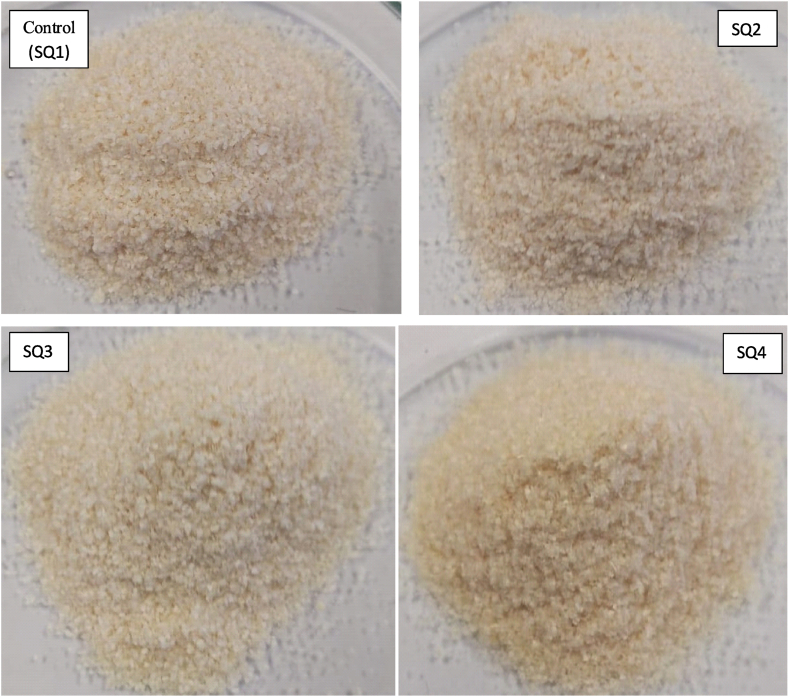
Chitosan from *M. rosenbergii*: SQ1, SQ2, SQ3 and SQ4 represents sequences 1, 2, 3 and 4 respectively.

In this study, chitosan was successfully extracted from shell waste of *M. rosenbergii* through chemical method by re-ordering the traditional sequence whereby four chitosan samples were obtained as shown in Fig. 2. Slight colour and texture variations were obvious and further characterization revealed more differences among the four chitosan samples.

### 3.1 Chitin and chitosan yield

The process conditions established for demineralization and deproteinization to obtain chitin in this study proved successful. Highest chitin yield of 23.99 ± 0.61% (11.99 g) was obtained in SQ4 which was significantly different from the rest of the sequences (Table 1). The general result of chitin yield across the sequences falls within the ranges reported in previous studies from crustaceans. For instance, 14–28% chitin yield has been reported from crab species [28], while 23.25% were recorded from mussel cuticle [29]. Similarly, Hubert Ribeiro & dos Santos, (2019) who recommended isolation of chitin and chitosan as a means of cleansing the environment of contaminants from crustacean waste, reported 15–20% chitin from shrimp shell waste.

**Table 1:** Physicochemical properties of chitosan from shell waste of *M. rosenbergii*.

Physicochemical properties	Sequence	*M. rosenbergii*	p. value
Chitin yield (g)			0.011
	Control (SQ1)	7.82 ± 1.59^b^	
	SQ2	8.26 ± 0.87^b^	
	SQ3	8.52 ± 0.70^b^	
	SQ4	11.99 ± 1.47^a^	
Chitosan yield (g)			0.801
	Control (SQ1)	6.82 ± 1.42^a^	
	SQ2	7.12 ± 0.87^a^	
	SQ3	7.16 ± 0.17^a^	
	SQ4	7.58 ± 0.85^a^	
Percentage chitosan yield (%)			0.838
	Control (SQ1)	14.31 ± 2.10^a^	
	SQ2	14.25 ± 1.74^a^	
	SQ3	14.19 ± 0.19^a^	
	SQ4	15.17 ± 1.69^a^	
Moisture (g)			0.228
	Control (SQ1)	0.09 ± 0.02^a^	
	SQ2	0.08 ± 0.01^a^	
	SQ3	0.06 ± 0.02^a^	
	SQ4	0.08 ± 0.01^a^	
Percentage moisture (%)			0.920
	Control (SQ1)	8.67 ± 1.53^a^	
	SQ2	8.01 ± 0.05^a^	
	SQ3	6.01 ± 2.00^a^	
	SQ4	8.00 ± 1.00^a^	
Ash (%)			0.001
	Control (SQ1)	19.66 ± 1.45^a^	
	SQ2	18.65 ± 1.30^a^	
	SQ3	12.46 ± 1.45^b^	
	SQ4	13.52 ± 1.61^b^	
FBC (%)			0.001
	Control (SQ1)	472.33 ± 4.93^d^	
	SQ2	584.27 ± 3.61^b^	
	SQ3	533.00 ± 7.63^c^	
	SQ4	629.33 ± 1.53^a^	
WBC (%)			0.001
	Control (SQ1)	482.33 ± 3.37^b^	
	SQ2	474.32 ± 2.52^b^	
	SQ3	512.00 ± 3.61^a^	
	SQ4	462.52 ± 5.03^c^	
Solubility (%)			0.014
	Control (SQ1)	60.21 ± 5.74^b^	
	SQ2	59.23 ± 7.52^b^	
	SQ3	62.36 ± 6.66^b^	
	SQ4	66.43 ± 2.61^a^	
DDA (%)			0.001
	Control (SQ1)	53.27 ± 5.91^b^	
	SQ2	54.87 ± 4.45^b^	
	SQ3	61.66 ± 4.28^b^	
	SQ4	80.67 ± 2.52^a^	

**Note:** Data presented as mean ± standard deviation. Means in the same column with different superscript differ significantly (*p ≤ 0.05*).

This study revealed that percentage chitosan yield is never affected by re-ordering the traditional sequence of chitosan production (Table 1). The general yield of chitosan in the present study fell below the 52.2% obtained from *Litopenaeus vannamei* using chemical and microwave methods [31]; and 17% from residue of *Catharsius molossus* L [10]. In another development, the yield of chitosan in this study was found higher than the 5.89% obtained from shell waste of freshwater crab, *Potamon algeriense* using a standardised and reversed chemical method (the traditional method) [9].

Over the years, many researchers have affirmed the hygroscopic nature of chitosan and established that the moisture content of commercial chitosan generally falls below 10%. Interestingly, the present study produced chitosan with desirable level of moisture content. Although SQ1 chitosan had the highest level of moisture content (8.67 ± 1.53%), it was found not significantly different from the rest of the sequences (Table 1) [26]. in their study reported chitosan moisture content of 0.35, 0.41 and 0.52% from shells waste of crab, squilla and commercial chitosan respectively. In addition [25], reported that chitosan from shrimp shells, fish scale and crab contain moisture content of 0.0004%, 0.009% and 0.0004% respectively. All these results, which were obtained using the conventional sequential protocol, fell far below the results of the present study. This study therefore submits that re-ordering the sequential steps in chitosan production does not affect the final moisture level of the product significantly.

In the chemical isolation of chitosan, a successful demineralization process can be measured by the level of the remaining ash after undergoing an anaerobic combustion process through a muffle furnace [32]. The higher the ash content, the less successful demineralization process and vice versa [33]. Many chemical solutions including sodium hypochlorate, potassium hydroxide, H_2_O_2_, alcohols etc have been used to remove pigments from raw shells of crustaceans during chitin and chitosan extraction. This has largely been done for aesthetic purposes. There is no special reason for the choice of ethanol for decolouration in the present study; However, it was revealed that decolouration of crude shell before demineralization enhanced hydrolysis of the shell in the later process, leading to more removal of minerals and proteins. This was evident in the lower ash content of the sequences beginning with Decolouration.

The relatively low ash content in the present study suggests that substantial levels of the inorganic materials in the raw shell of *M. rosenbergii* were removed by the acid applied. It was however evident that SQ3 (12.46 ± 1.45%) and SQ4 (13.52 ± 1.61%) which began with decolouration had the lowest ash content which were not significantly different from each other (Table 1). The above result disagrees with that of [22] who reported 0.12–0.86% and 0.19–0.51% ash content from crayfish shell using the conventional protocol. In another development, ash content of chitosan from fish scale, crab and shrimp shell were reported as 1%, 2.5% and 0.03% respectively [25]. It can be deduced from the result of the ash content that the decolouration process which removed pigments from the raw shell further prepares it for better hydrolysis and reagent penetration during the subsequent steps.

The water binding capacity (WBC) of a hydrophilic substance refers to its ability to associate favourably with water while fat binding capacity (FBC) expresses the quantity of absorbed oil per unit weight of the substance [34,35]. Studies have shown that WBC of chitosan is highly influenced by the levels of demineralization, deproteinization and deacetylation reactions. This by extension implies that the nature of the acid as well as the alkali utilized in addition to their concentration and ratio of mixture with the solute to achieve the above processes must be carefully chosen in order to achieve expected WBC. Commercial chitosan has been reported with WBC of 812.67 ± 7.64% [3] having compared with 1095.66 ± 6.03, 1270 ± 11, and 1161.67 ± 10.37% recorded by another study [34]. In addition [36], studied on the chemical extraction of chitosan by applying the conventional sequence to obtain chitosan with WBC of 652 ± 0.02% which was less than that of commercial chitosan. In the study under consideration, the highest WBC was recorded in SQ3 (512.00 ± 3.61%) while the least was obtained from SQ4 (462.52 ± 5.03%) which were all significantly different from SQ1 and SQ2 (Table 1). It is worthy of note that the change in the sequence of production had impacted significantly on the processes leading to the final product, thereby accounting for the variability in the textural composition of the final chitosan product. Similarly, the measure in the level of oil absorbed by chitosan, simply referred to as the FBC in the present study differ significantly across the sequences due to the uneven nature of the resultant chitosan particles [35]. The highest FBC of chitosan was recorded from SQ4 (629.33 ± 1.53%) which was close to that of commercial chitosan (676.2 ± 43.38%).

### 3.2 Solubility and DDA of chitosan

Irrespective of sequence, chitosan produced in the present study was insoluble in water with pH above 7.0. The likely reason for this occurrence is that the amino groups in the chains of chitosan are readily deprotonated in alkaline solution [37]. Therefore, chitosan solubility was tested in 1% acetic acid solution where SQ4 chitosan recorded the highest value (66.43 ± 2.61%); significantly different from all other sequences under study (Table 1). Chitosan possesses d-glucosamine unites carrying mobile amino groups acquiring positively charged ions which are responsible for the all-important properties of the biopolymer such as solubility and antimicrobial property [1]. The deprotonation of the amino groups in acidic solution is of paramount importance as far as chitosan application is concerned. In another study, solubility of chitosan from squilla was established at 98% which was close to 98.9% from commercial chitosan [26] and higher than what was obtained in the present study. It is important to highlight at this point that many factors such as concentration of the acid and alkali, duration of reaction, size of the product all greatly influence solubility of the final chitosan product [35]. The low solubility of chitosan in the present study is as a result of incomplete demineralization of the raw shell, leading to higher residual minerals in the chitosan. This is evident in the higher level of ash in the chitosan [32]. Ref. [38] further corroborated this submission and added that high chitosan solubility means the presence of free amine groups that can be easily deprotonated in the presence of aqueous solutions.

DDA of chitosan entails the extent of the expulsion of acetyl chains from the chitin molecular chains and the corresponding addition of amino groups. The level of reactive amino groups present in the structure of chitosan determines its level of application [39]. posited that chitosan is only made useful and referred as such when its DDA is recorded above 70%. In the study under consideration, re-ordering the conventional sequence of chitosan production had little influence on the DDA of the resultant chitosan. However, the study showed that adopting the sequential arrangement whereby decolouration precedes demineralization before deproteinization resulted to better deacetylation process; culminating to chitosan with better DDA. Chitosan from SQ4 (80.67 ± 2.52%) which was significantly different from the rest of the sequences clearly shows better prospects than 70.0–78.3 mol% reported from black soldier flies [40] and similar to 70.66%–80.88% recorded from a fungal source [41].

### 3.3 Bulk, tapped densities, compressibility, Hausner ratio (HR) and Carr's index (CI)

In this study, bulk (BD) and tapped (TD) densities as well as compressibility, Hausner ratio and Carr's index were studied in order to understand the physical properties of the isolated chitosan. Technically, BD explains the ability of a powder to undergo compression and compaction. These particular properties are often taken into account when identifying drug fillers in pharmaceuticals. Unfortunately, chitosan is least considered for filler binders in the midst of other diluents due to low BD and TD [42]. It has been confirmed in this study that chitosan possesses low BD and TD irrespective of the order of the production sequences (Table 2). There was no significant difference, the sequence notwithstanding. The low BD and TD may be as a result of high particulate irregularities leading to a chitosan of porous nature [43]. It was evidently clear from the statistical results of this study that re-ordering the sequence of production of chitosan does not improve the compressibility and HR of chitosan while little variations occurred in the CI. The results of Hausner ratio in this study were above 1.137 and 1.125 obtained by Ref. [44] in chitosan from *Mercenaria mercenaria*. The relatively higher Hausner ratio further confirms that the chitosan produced in the present study have poor flowability [45]. In addition, CI value has been reported to providing an information to the level of flowability and by extension, the compressibility of chitosan. In this manner, the CI numbers of 10, 12–16, 18–21, and 23–28 represents excellent, good, fair, and poor flow properties of chitosan accordingly [43]. Therefore, the chitosan in this study with CI value of 31–35 should be considered as one of poor flowability and compressibility.

**Table 2:** Physical properties of chitosan extracted from shell waste of *M. rosenbergii*.

Physical properties	Sequence	*M. rosenbergii*	p. value
Bulk density (g/cm^−3^)			0.448
	Control (SQ1)	0.20 ± 0.02^a^	
	SQ2	0.20 ± 0.01^a^	
	SQ3	0.22 ± 0.02^a^	
	SQ4	0.21 ± 0.01^a^	
Tap density (g/cm^−3^)			0.453
	Control (SQ1)	0.31 ± 0.03^a^	
	SQ2	0.29 ± 0.02^a^	
	SQ3	0.29 ± 0.02^a^	
	SQ4	0.30 ± 0.01^a^	
Compressibility			0.142
	Control (SQ1)	34.00 ± 2.00^a^	
	SQ2	30.83 ± 3.63^a^	
	SQ3	28.60 ± 2.63^a^	
	SQ4	30.31 ± 1.12^a^	
Hausner ratio (HR)			0.109
	Control (SQ1)	1.51 ± 0.05^a^	
	SQ2	1.46 ± 0.06^a^	
	SQ3	1.38 ± 0.07^a^	
	SQ4	1.45 ± 0.05^a^	
Carr's index (CI)			0.001
	Control (SQ1)	35.08 ± 0.53^a^	
	SQ2	31.70 ± 2.93^a^	
	SQ3	24.50 ± 1.39^b^	
	SQ4	30.79 ± 2.26^a^	

**Note:** Data presented as mean ± standard deviation. Means in the same column with different superscript differ significantly (*p* ≤ *0.05*).

### 3.4 Percentage (chitin yield, inorganic material, protein and pigment)

It has been established that raw shells of crustaceans are made up of chitin, proteins, pigments and inorganic mineral materials such as calcium carbonate and phosphates [46]. This implies that chemical isolation of chitosan from the crustacean shell would require elimination of all other elements and finally deacetylating the chitin into chitosan. The present study attempted to analyse the effect of re-ordering the conventional sequence on the percentage yield of chitin, and removal of protein, pigment as well as inorganic material. It was revealed from the analyzed results that variation in the sequential steps had resulted to a significant difference (*p* ≤ *0.05*) in the percentage levels of chitin, inorganic compounds, protein and pigment as shown in Table 3. The highest chitin yield of 23.99 ± 0.61% (SQ4) was higher than the 4.05 ± 0.85% and 4.2% obtained from shell waste of *S. hextii* and *S. indica* respectively using the conventional method [47]. Chitin is associated with over 200 applications and mostly applied in its alkaline hydrolysed form called chitosan. Due to their numerous applications, many attempts have been made in identifying their sources and conscious efforts made for large scale production [9]. reported that the general chitin level of crustaceans falls within the range of 20–30% which falls within the value obtained in the present study. In another development [28], posited that using the conventional method of chemical extraction, 14–28% yield of chitin was realized from shell waste of crab. The high yield of chitin from SQ4 in this study may be as a result of the more efficient process of demineralization after decolouration. It is believed that the removal of pigment before demineralization must have further prepare the substance for better hydrolysis and better penetration of acid to remove the adherent inorganic materials.

**Table 3:** Percentage chemical composition of *M. rosenbergii* shell based on sequence of extraction.

Parameter	Sequence	*M. rosenbergii*	p. value
Percentage chitin level (%)			0.008
	Control (SQ1)	15.97 ± 2.63^b^	
	SQ2	16.53 ± 1.74^b^	
	SQ3	17.05 ± 2.93^b^	
	SQ4	23.99 ± 0.61^a^	
Percentage protein removal (%)			0.001
	Control (SQ1)	19.67 ± 0.97^b^	
	SQ2	36.28 ± 5.90^a^	
	SQ3	31.88 ± 2.05^a^	
	SQ4	40.20 ± 2.94^a^	
Percentage inorganic removal (%)			0.017
	Control (SQ1)	37.49 ± 1.34^b^	
	SQ2	34.38 ± 8.05^ab^	
	SQ3	25.12 ± 1.64^b^	
	SQ4	40.36 ± 3.80^a^	
Percentage pigment removal (%)			0.001
	Control (SQ1)	5.13 ± 1.23^d^	
	SQ2	12.81 ± 2.46^c^	
	SQ3	26.80 ± 1.70^a^	
	SQ4	18.85 ± 2.34^b^	

**Note:** Data presented as mean ± standard deviation. Means in the same column with different superscript differ significantly (*p ≤ 0.05*).

As earlier stated, raw shell of crustaceans contains proteins and inorganic minerals in addition to chitin. To remove proteins, hydroxide (NaOH) was applied while hydrochloric acid (HCl) was utilized to achieve demineralization. In all these, the effect of re-ordering the conventional sequence was examined. Studies have shown that the carapace of crustaceans possesses up to 30–50% calcium carbonate and 30–40% protein [9]. In addition [31], in their extraction of chitin and chitosan from shrimp shell using the conventional method reported 25–40% protein and 40–55% calcium carbonate. The results of the present study were found in agreement with previous studies.

### 3.5 FTIR

**Fig. 3:**
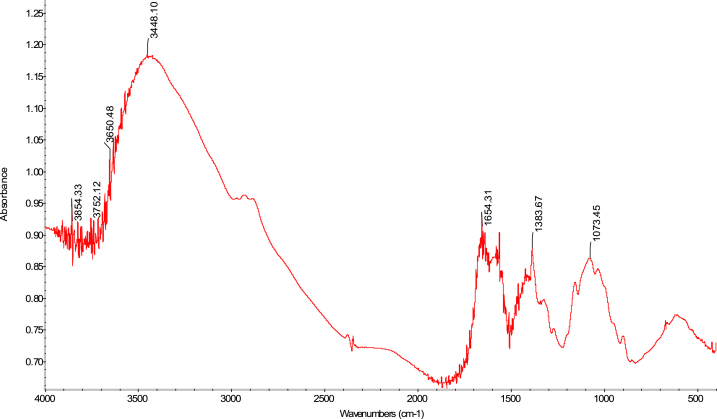
FTIR spectra of commercial chitosan.

**Fig. 4:**
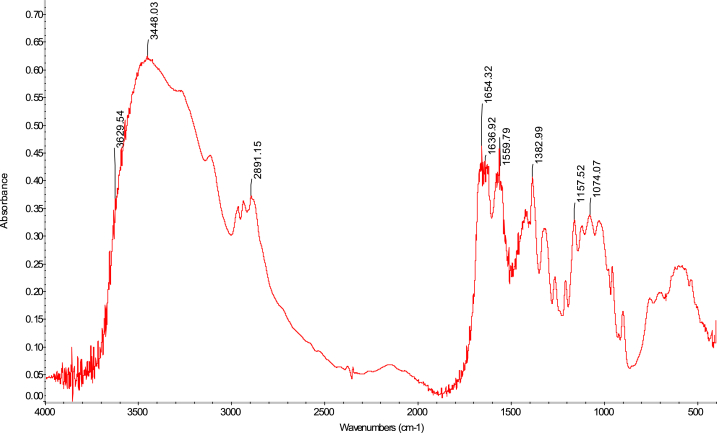
FTIR spectra of control (SQ1) chitosan extracted from *M. rosenbergii*.

**Fig. 5:**
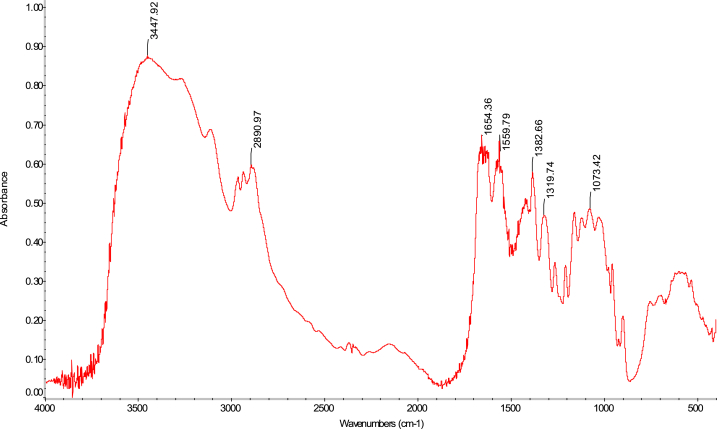
FTIR spectra of SQ2 chitosan extracted from *M. rosenbergii*.

**Fig. 6:**
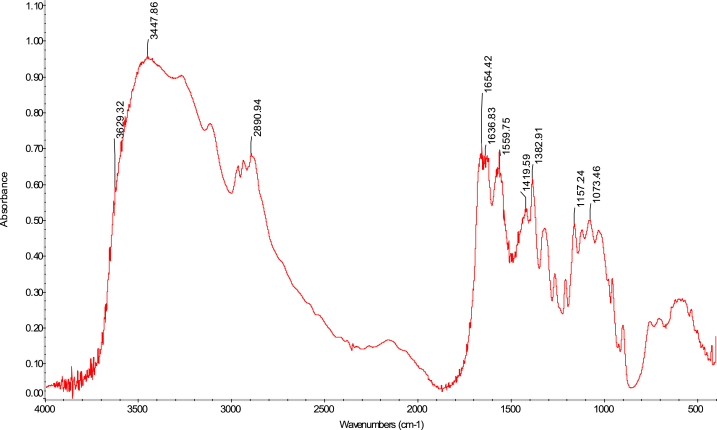
FTIR spectra of SQ3 chitosan extracted from *M. rosenbergii*.

**Fig. 7:**
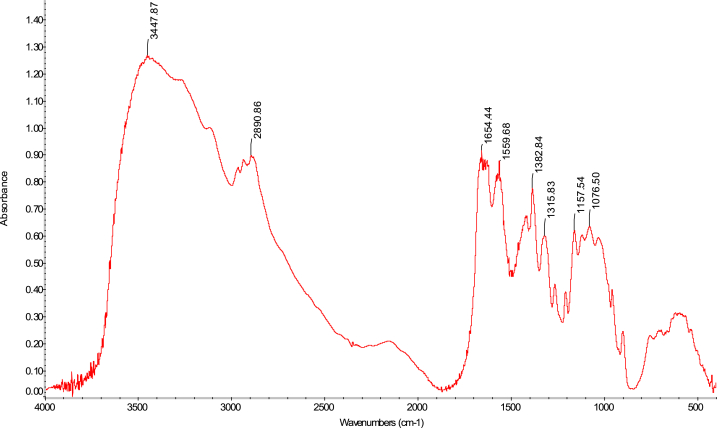
FTIR spectra of SQ4 chitosan extracted from *M. rosenbergii*.

The FTIR analytical approach was used to pinpoint the precise functional groups in the chitosan samples. In terms of absorbance and wavelength, the spectra of the chitosan derived from *M. rosenbergii* biowaste were found to be identical to those of commercial chitosan (F. 32), with some minor variations from one sequence to the next. In general, success in the deacetylation process was indicated by a distinct weakening of the band above 1600 cm^−1^ (C=O), which was seen across all sequences (Fig. 43–76) [48,49]. The broad band OH and NH_2_ maximum stretching FTIR spectra results in the current study were higher (3447 cm^−1^) than those from shrimp shell chitosan (3200-3374 cm^−1^) by Refs. [[48], [49], [50]]. In addition, Table 4 demonstrates that the modification in the order of chitosan manufacture had no impact on the NH_2_ and OH stretching, (C=O) secondary amides stretching, (NH_2_) bend, CH bend, and (CH_3_) symmetrical deformation, as well as the C–O–C) asymmetric stretch in the phase ring [48]. In chitosan produced by kGy irradiation of crab shells, these functional groups exhibited similar behaviour [47]. The amide III peaks in the chitosan molecule are said to be centred around 1320 cm^−1^ [47,48,51]. In the present study, amide III was recorded at 1382 cm^−1^, 1382 cm^−1^, 1382 cm^−1^ and 1382 cm^−1^ for SQ1, SQ2, SQ3 and SQ4 respectively where highest bands were observed in SQ1. The vibration bands observed across sequences were similar to that of commercial chitosan (Fig. 3). The amide III peak shows the presence of n acetyl glucosamine in the chitosan molecules produced [52]. Generally, the FTIR spectra results of chitosan from biowaste of *M. rosenbergii* as presented in this study does not show great disparity from that of commercial chitosan. Previous studies have shown that chitosan with highly compact nature possess strong hydrogen bonding and usually of the α-form while β-forms are differentiated by their highly soluble and reactive nature [53]. In the present study, the sharp absorbance and splitting of amide I band around 1654 cm^−1^ shows that chitosan extracted from *M. rosenbergii* is of the α-form [[54], [55], [56]] (see Fig. 4, Fig. 5, Fig. 6, Fig. 7).

**Table 4:** Functional groups of chitosan sourced from *M. rosenbergii*.

Functional group	Sequence/vibration wavelength (cm^−^^1^)					Range of wavelength
	Commercial chitosan	Control (SQ1)	SQ2	SQ3	SQ4	
NH_2_ and OH stretching	3448	3448	3447	3447	3447	3650–3400
(CH_3_) symmetric stretch and (CH_2_) asymmetric stretch	2869	2891	2890	2890	2890	2919–2868
(C=O) secondary amides stretch (Amide I)	1654	1654	1654	1654	1654	1650–1550
(NH_2_) bend, C–N stretch	1550	1559	1559	1559	1559	1560–1500
Amide III	1383	1382	1382	1382	1382	1390–1370
Aromatic band (C–O)	1303		1319		1315	1310–1250
(C–O–C) asymmetric stretch in phase ring	1073	1074	1073	1073	1076	1124–1087
(C–O–C) asymmetric stretching	1027	1157		1157	1157	1124–1087

### 3.6 Orientation of the crystalline nature (XRD diffraction) of raw shell, commercial, and *M. rosenbergii* chitosan

**Fig. 8:**
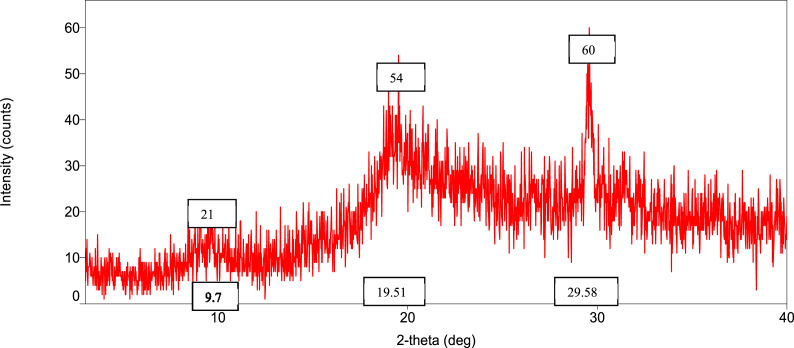
XRD graph from raw powder shell of *M. rosenbergii*.

**Fig. 9:**
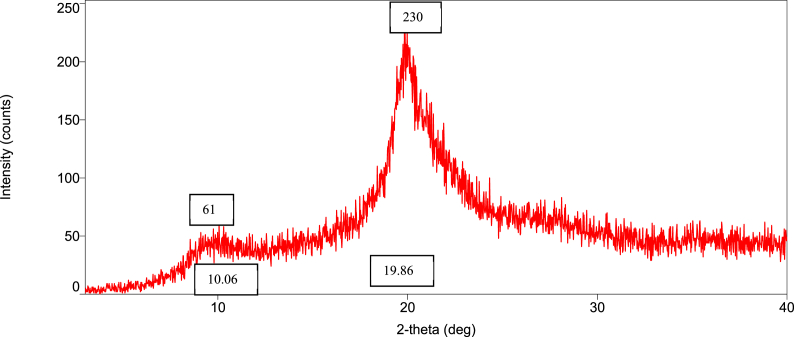
XRD graph of commercial chitosan.

**Fig. 10:**
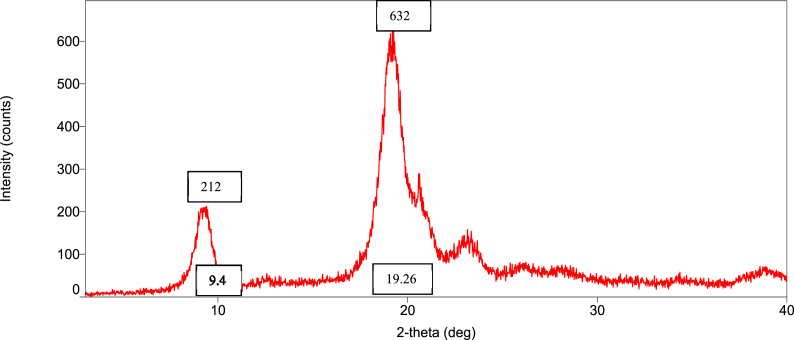
XRD graph of Control (SQ1) chitosan from *M. rosenbergii*.

**Fig. 11:**
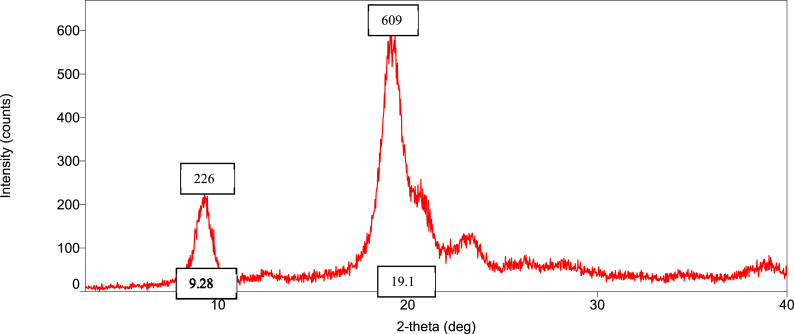
XRD graph of (SQ2) chitosan from *M. rosenbergii*.

**Fig. 12:**
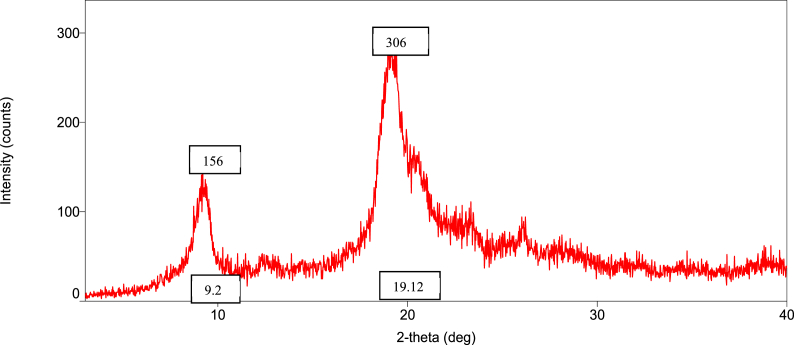
XRD graph of (SQ3) chitosan from *M. rosenbergii*.

**Fig. 13:**
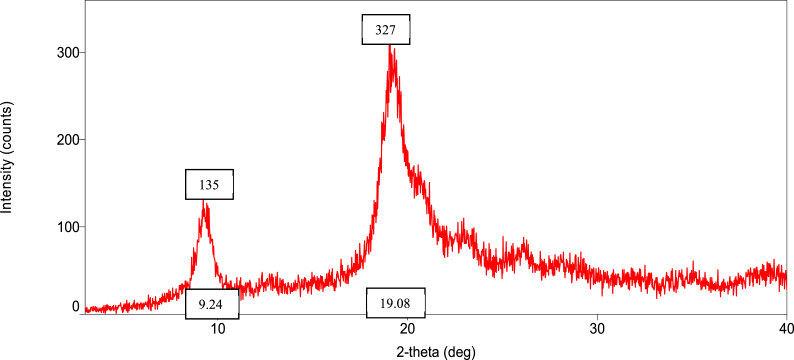
XRD graph of (SQ4) chitosan from *M. rosenbergii*.

The diffraction intensities and crystallinity index (CrI) values of powdered raw shell *M. rosenbergii* and chitosan from four different sequences are presented in Fig. 8, Fig. 9, Fig. 10, Fig. 11, Fig. 12, Fig. 13. The intensities of the *M. rosenbergii* raw powdered shell recorded peaks at higher diffraction angles (9.7° and 19.51°). More so, an extra peak was also observed at 2θ value of 29.58°. The raw powder shell utilized in this study yielded an overall lower CrI of 70.37%. The diffraction intensity of chitosan from *M. rosenbergii* followed a pattern where the highest peaks were observed in SQ1 (632) and SQ2 (609) at 2θ values of 19.26° and 19.1°, respectively. The highest CrI values were recorded from SQ2 (91.46%) while the lowest was obtained in SQ4 (83.43%). *M. rosenbergii* chitosan from all sequences recorded CrI higher than that of commercial chitosan. The high diffraction intensity and crystallinity of the planes suggested properties of polymorphic α chitosan [57].

In a review of the uncooked shell Using XRD, *M. rosenbergii* demonstrated that the rhombohedra calcite has the highest diffraction intensity. In this investigation, the raw shell of *M. rosenbergii* had a lower diffraction angle than the shell of *Potamon algeriense*, which had a diffraction angle of 2θ = 29.4° [9]. Magnesium and chitin, which make up the crystalline portion of the powder shell, were visible in the strong calcite [1] as well [1,9]. There have been two crystal forms of chitosan described. These are alluded to as either monoclinic system forms I or the monoclinic system form II, where 2θ of the former is about 10° and the latter is around 20° [10]. The 2θ in chitosan's form I and form II in the current investigation is consistent with the results that have been published. Although all sequences of chitosan showed somewhat lower angles, the values nevertheless matched those seen in crab and squilla [10,58].

The degree of deacetylation of chitosan can be used to determine its crystallinity. If 100% DDA is attained, chitosan is thought to be completely crystalline; however, any state with some acetylation is thought to be semi-crystalline [38]. As demonstrated in Table 5, there was variance in the XRD spectrum intensity among the sequences in the current investigation, demonstrating that altering the chitosan production sequence had some structural effects on the finished product. The chitosan made from *M. rosenbergii* may have preserved unreacted chitin, which could explain why crystallites appeared along amorphous areas [38,59]. The overall CrI of the chitosan under investigation was high; it was greater compared to commercial chitosan. The high CrI could be a result of the adequate liquid to particle ratio, which allowed alkali and acid to adequately penetrate the crystallite of raw shell and chitin [35]. The values of CrI were found to be greater than the 82.30% reported by Ref. [60] from *M. rosenbergii* and similar to those obtained from crab, shrimp, and fish scale chitosan by Refs. [25,35,60]. According to Ref. [61], determination of the crystalline value of chitosan is sacrosanct, and further added that lower crystalline structure chitosan performs better in wastewater treatment. This suggests that chitosan from SQ4, of *M. rosenbergii* has better wastewater coagulation properties.

**Table 5:** Crystallinity index of extracted chitosan.

Source/sequence	Crystallinity index (%)
*M. rosenbergii* raw powder shell	70.37
Commercial chitosan	72.96
SQ1 chitosan	91.28
SQ2 chitosan	91.46
SQ3 chitosan	87.01
SQ4 chitosan	83.43

### 3.7 Scanning electronic microscope (SEM) images of chitosan extracted using four sequences from *M. rosenbergii* raw shell

**Fig. 14:**
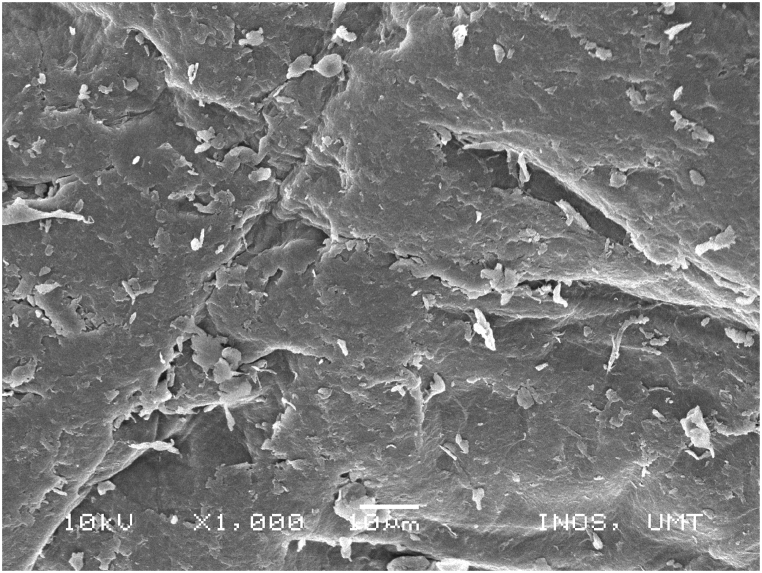
SEM images of commercial chitosan.

**Fig. 15:**
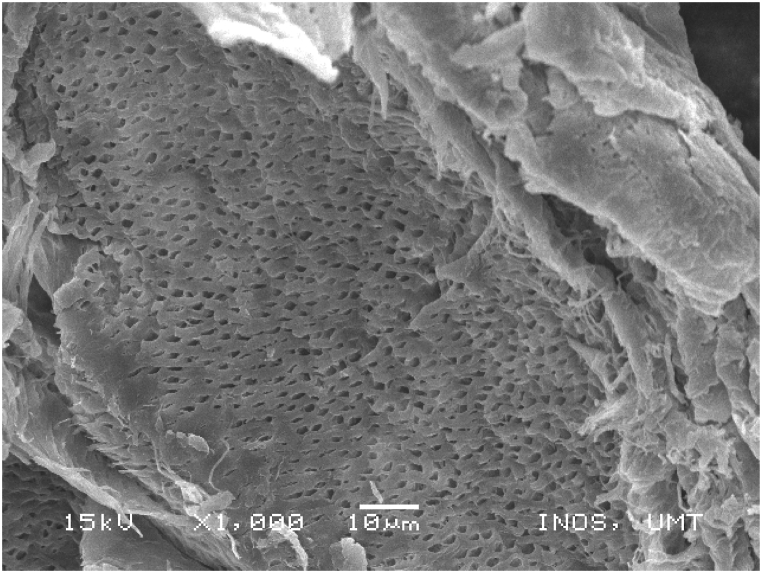
SEM image of control (SQ1) chitosan from *M. rosenbergii*.

**Fig. 16:**
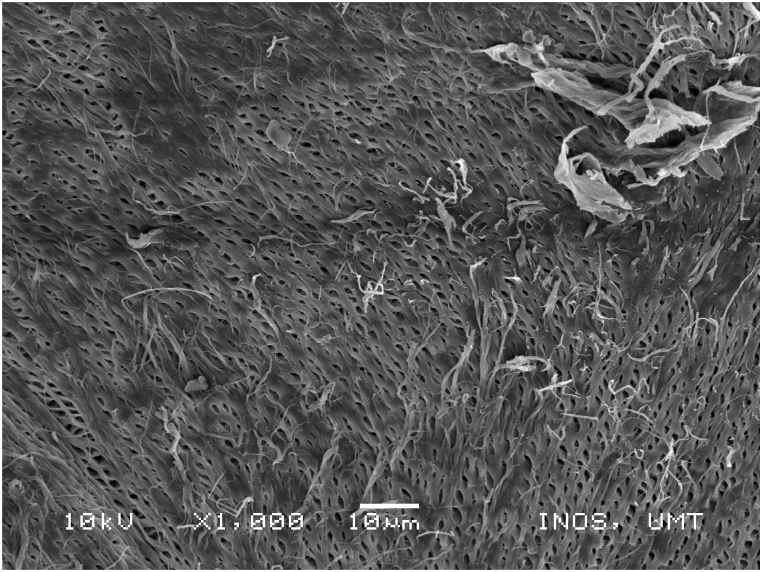
SEM image of SQ2 chitosan from *M. rosenbergii*.

**Fig. 17:**
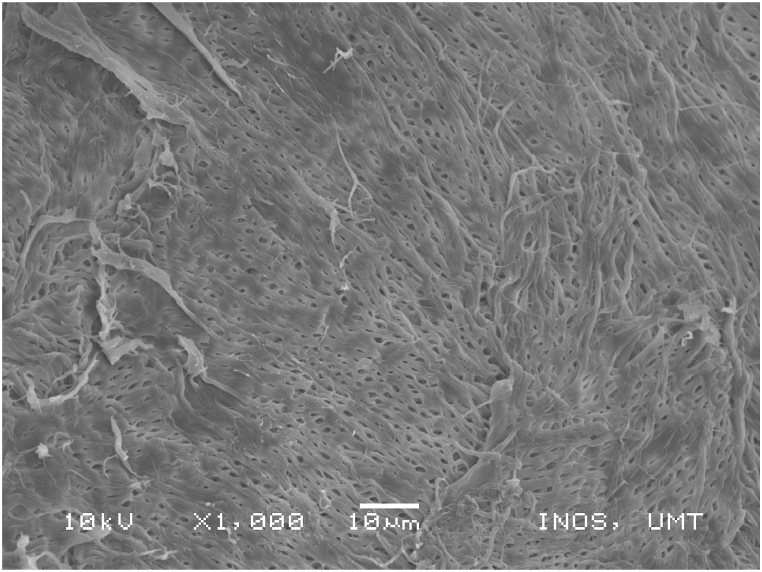
SEM image of SQ3 chitosan from *M. rosenbergii*.

**Fig. 18:**
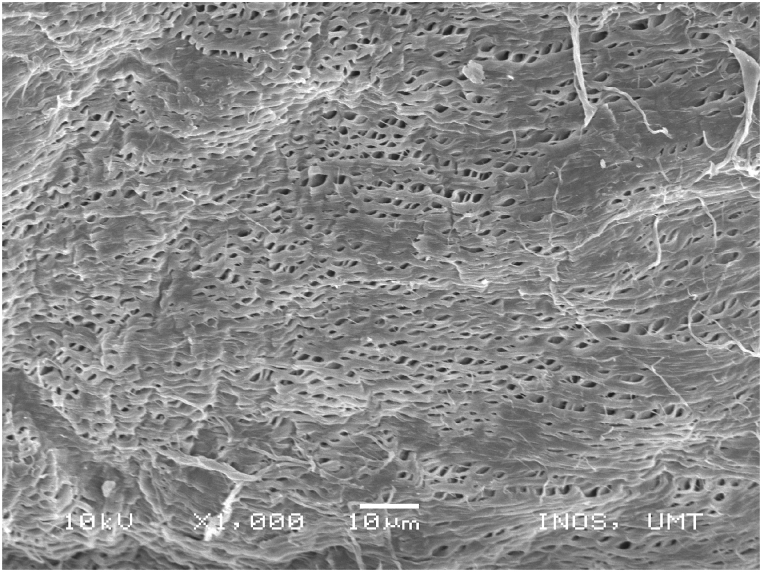
SEM image of SQ4 chitosan from *M. rosenbergii*.

SEM also offers helpful details on the morphologies and microstructures of the chitosan, in addition to the use of XRD to ascertain the structural composition and distinction between chitosan from the source and production processes under examination. SEM is a method for creating images by scanning the surface of chitosan particles with a concentrated electron beam. The images employed in the study were created by the interaction of the particle's atoms and electrons [50]. The SEM images of chitosan from commercial source (Fig. 14) and *M. rosenbergii* showed a conspicuously similar rough structure but different from the findings of [62] using the conventional sequence of chitosan extraction. The sponge and leaf-like morphology of chitosan from *M. rosenbergii* (Fig. 15, Fig. 16, Fig. 17, Fig. 18) closely resembled that obtained from crab and squilla chitosan [13,25,63].

The effective use and application of chitosan can be determined from its surface morphology properties. Therefore, chitosan from *M. rosenbergii* could be fit for application in textile industry [38]. At 1000× magnification, chitosan obtained showed only little variation across the four sequences but largely comparable with previous studies such as porous chitosan from commercial source reported by Ref. [61]. In addition, the porous and fibril structure of *M. rosenbergii* chitosan closely resembles that reported from fish scale [49]. Specifically, the chitosan of this nature could also be useful in tissue engineering as well as textile application [35].

## 4 Conclusion

In this study, chitin and chitosan were successfully isolated using the chemical method. The alteration of the chemical traditional sequence of chitin and chitosan production had significant effects on the resultant yield and by extension, the physicochemical characteristics. It was observed that sequence four (SQ4) which involves the decolouration of the dry powder shell, followed by demineralization and deproteinization before deacetylation yielded higher chitin (23.99%) and chitosan (15.17%). Furthermore, results of the characterization of the chitosan obtained from the four sequential arrangements showed that chitosan from SQ4 had better physical and chemical properties. The present study reveals t higher DDA (80.67%) and solubility (66.43%) of chitosan from *M. rosenbergii* from SQ4. Generally, chitosan across sequences had low bulk and tapped densities which suggested their porous nature. The porous nature was further corroborated by SEM images and confirmed that isolated chitosan agreed with commercial chitosan in terms of morphological appearance. In addition, the FTIR spectra of the isolated chitosan revealed a sharp absorbance and splitting of amide I band at 1654 cm^−1^ thereby confirming that chitosan was of the α-form. The porous and fibril nature of the chitosan produced in this study could find useful application in tissue engineering and textile industry.

## Author contribution statement

Nor Azman Kasan and Siti Rozaimah Sheikh Abdullah: Conceived and designed the experiments; Contributed reagents, materials, analysis tools or data.

Benedict Terkula Iber: Performed the experiments; Analyzed and interpreted the data; Wrote the paper.

Donald Torsabo: Analyzed and interpreted the data; Contributed reagents, materials, analysis tools or data.

Che Engku Noramalina Che Engku Chik: Performed the experiments; Wrote the paper.

Hassimi Abu Hassan and Fachrul Wahab: Performed the experiments; Analyzed and interpreted the data.

## Funding statement

Nor Azman Kasan was supported by 10.13039/501100003093Ministry of Higher Education, Malaysia [LRGS/2018/USM-UKM/EWS/01]. Higher Institution Centre of Excellence (HICoE), 10.13039/501100023351Institute of Tropical Aquaculture and Fisheries (AKUATROP) [Vot. No. 63933, JPT.S(BPKI) 2000/016/018/ 015 Jld.3 (23) and Vot. No. 56050, UMT/PPPI/2-2/5 Jld.2 (24)].

## Data availability statement

Data will be made available on request.

## Declaration of interest's statement

The authors declare no competing interest
